# The Awaji criteria increases the diagnostic sensitivity of the revised El Escorial criteria for amyotrophic lateral sclerosis diagnosis in a Chinese population

**DOI:** 10.1371/journal.pone.0171522

**Published:** 2017-03-01

**Authors:** Da-Wei Li, Mingsheng Liu, Bo Cui, Jia Fang, Yu-Zhou Guan, Qingyun Ding, Xiaoguang Li, Liying Cui

**Affiliations:** 1 Department of Neurology, Peking Union Medical College Hospital, Chinese Academy of Medical Sciences, Beijing, China; 2 Neurosciences Center, Chinese Academy of Medical Sciences, Beijing, China; Westmead Hospital, University of Sydney, AUSTRALIA

## Abstract

**Objectives:**

The accurate and early diagnosis of amyotrophic lateral sclerosis (ALS) is important for extending the life expectancy of patients. However, previous studies that have assessed the diagnostic sensitivities of the Awaji criteria (AC) and the revised El Escorial criteria (rEEC) in patients with ALS have been inconsistent, most of them were consensual regarding the advantage of Awaji over conventional criteria. Our study sought to compare the roles of AC and rEEC in the diagnosis of ALS.

**Methods:**

Data from a total of 294 consecutive patients with ALS were collected between January 2014 and August 2015 in the Peking Union Medical College Hospital. The clinical and electrophysiological records of 247 patients were eventually analyzed. The primary outcome measures were the sensitivities of the AC and rEEC for the diagnosis of ALS.

**Results:**

The sensitivity of probable or definite ALS as diagnosed with the AC (78%) was greater than that of the rEEC (36%, *P* <0.001). Following the application of the AC, 103 of the 147 patients categorized as probable ALS-laboratory supported from the rEEC were upgraded to probable or definite ALS, and 44 were downgraded to possible ALS.

**Conclusions:**

Our data demonstrated that the AC exhibited greater diagnostic sensitivity than the rEEC in a Chinese ALS population. The use of the AC should be considered in clinical practice.

## Introduction

Amyotrophic lateral sclerosis (ALS) is a fatal neurodegenerative disorder that is characterized by progressive muscle weakness, atrophy and upper motor neuron (UMN) signs[1, 2]. Seventy percent of patients have a survival rate of less than 3 years [3]. Accurate and early diagnosis plays an important role in extending the life expectancy [4, 5].

The diagnosis of ALS relies on the presence of a combination of UMN and lower motor neuron (LMN) signs in the same specific body regions [1, 6]. Two diagnostic criteria are currently used in the diagnosis of ALS, i.e., the revised El Escorial criteria (rEEC) and the Awaji criteria (AC) [6, 7]. The rEEC is primarily used in clinical trials, but it has been reported that the rEEC delays the diagnosis and grading of ALS [8]. To increase the diagnostic sensitivity for ALS, the AC was developed and recommends that electromyographic (EMG) abnormalities should be taken as equivalent to lower motor neuron abnormalities, and clinical examination findings and fasciculation potentials are regarded as evidence of acute denervation in the presence of chronic neurogenic changes [7].

However, comparisons between the diagnostic sensitivities of the AC and rEEC in patients with ALS remain inconsistent. Most of them have reported that the AC was obviously more sensitive than the rEEC [9–12]. For example, Carvalho et al (2009) stated that the AC was obviously more sensitive that the rEEC in 55 cases of ALS [9]. Other studies have found no significant differences between the AC and rEEC in the diagnosis of ALS [13, 14]. For example, Gawel et al (2014) reported no significant difference between the AC and rEEC in the diagnosis of ALS in 135 ALS cases [14]. The reasons for this inconsistency may include differences in study populations and methodological limitations [15]. A potential methodological limitation is that the performance of EMG did not meet the AC requirements [16, 17]. The AC requires the assessment of at least two muscles in the cervical (upper limbs) and lumbosacral (lower limbs) regions, and one muscle in bulbar and thoracic paraspinal region [7]. Moreover, the diagnostic sensitivities of the AC and rEEC have not been examined in a Chinese population. The study should be performed in the Chinese population, because there is some heterogeneity in the age of disease onset, median survival time and genetic basis of ALS between Chinese and Western cohorts[18]. Peking Union Medical College Hospital is a center for diagnosis and treatment of severe and complicated diseases in China, and our group focuses on ALS diagnosis and treatment [19–21]. We have large numbers of ALS patients across the country, and we conducted a study to compare the diagnostic sensitivities of the AC and rEEC based on a detailed patient database.

The aim of the present study was to compare the diagnostic sensitivities of the AC and rEEC by reviewing the detailed clinical and neurophysiological data from a Chinese population.

## Material and methods

### Participants

We performed a retrospective analysis of prospectively consecutive acquired clinical and neurophysiological data from patients who attended our outpatient clinic for ALS at the Peking Union Medical College Hospital between January 2014 and August 2015. We included patients who fulfilled the rEEC for at least possible ALS at the initial investigation. Patients without EMG examinations in our laboratory were excluded. This study was approved by the Research Ethics Committee of Peking Union Medical College Hospital. The requirement for informed consent was waived due to the retrospective nature of the study.

### Diagnostic categorization

In this study, the rEEC was used to categorize the patients into four levels of diagnostic probability: clinically possible ALS, clinically probable ALS-laboratory supported, clinically probably ALS, and clinically definite ALS. The AC was used to divide the patients into clinically possible ALS, clinically probable ALS and clinically definite ALS [6, 7]. The details of the criteria categories of the rEEC and AC have been described in previous reports [6, 7, 16].

To categorize the two criteria, the clinical notes were examined. The clinical signs were recorded as positive or negative for lower motor neuron and/or upper motor neuron signs in each area of four anatomical regions that included the cranial, cervical, thoracic and lumbosacral regions. We also reviewed the clinical, neurophysiological, laboratory and neuroimaging data to exclude potential ALS mimic disorders.

### Neurophysiological examination

The neurophysiological examinations were reviewed to identify lower motor neuron abnormalities. Needle EMG examinations were performed following a standardized protocol on both sides muscles that included sternocleidomastoid, abductor digiti minimi muscle of the hand, extensor digitorum communis, deltoideus, mid-thoracic paraspinal, tibialis anterior muscles and vastus medialis using a Viking IV EMG machine (Nicolet Biomedical, Madison, Wisconsin, USA). In each muscle, at least 10 insertions were performed to search for fibrillation potentials and positive sharp waves during complete muscle relaxation. Additionally, motor and sensory nerve conduction studies were performed in a minimum of median nerve, ulnar nerve, tibial nerve, and peroneal nerve. Muscle in completely or nearly completely relaxed was observed for at least 60s to identify fasciculation potentials, which was defined as spontaneous motor unit potentials at an irregular frequency[7]. The time interval between the patients’ clinical examinations and the EMGs never exceeded 1 week.

In this study, the patients’ clinical and EMG data were reviewed jointly by both investigators, and the diagnostic categories for each patient decided by consensus.

### Definition of sensitivity and the reference standard

For the purpose of calculating the sensitivities, if the criteria diagnoses were clinically definite ALS or clinically probable ALS, the patients were regarded as having a ‘positive ALS criteria diagnosis’. Conversely, if the criteria diagnoses were clinically probable laboratory-supported ALS or clinically possible ALS, the patients were assigned to the ‘negative ALS criteria diagnosis’ category [22]. The reference standard or gold standard that was used to confirm the diagnosis of ALS was disease progression during follow-up for at least six months as determined by history or examination [6, 23].

### Statistical analysis

The statistical analyses were performed using SPSS 17.0 (IBM Corporation, Chicago, IL, USA). The primary outcome measures were the sensitivities of the AC and rEEC in the diagnosis of ALS. McNemar’s test was used to determine the statistical significance of the sensitivity differences in paired binomial proportions. *P* <0.05 was considered as statistically significant.

## Results

**Fig 1:**
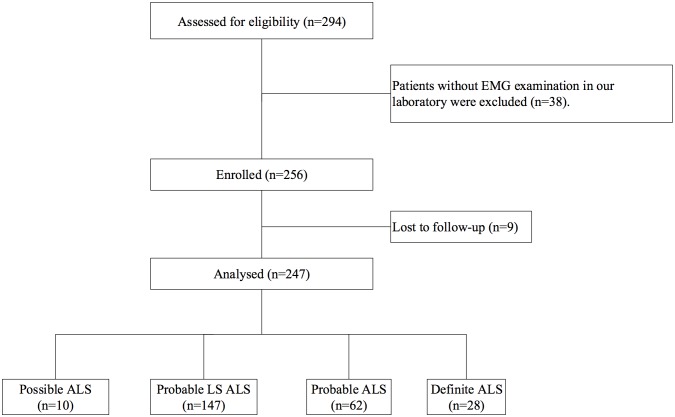
Flow diagram of the study. The clinical and electrophysiological records of 247 patients were eventually analyzed. ALS: amyotrophic lateral sclerosis; Probable LS: probable laboratory-supported.

Of the 294 consecutive outpatients, 38 were excluded because the EMG was not performed in our hospital, and 9 were lost to follow-up. The clinical and electrophysiological records of 247 patients were eventually analyzed (Fig 1). We included 139 men and 108 women with the mean (SD) age of 53.4 (11.1) years at assessment. The mean (SD) age at onset was 52.2(11.1)years and the mean (SD) disease duration was 15.5 (9.7) months. The number of symptom onset in bulbar, upper extremities and lower extremities were 46, 134 and 67, respectively. The mean and standard deviation of the revised amyotrophic lateral sclerosis functional rating scale score were 40±4.

**Fig 2:**
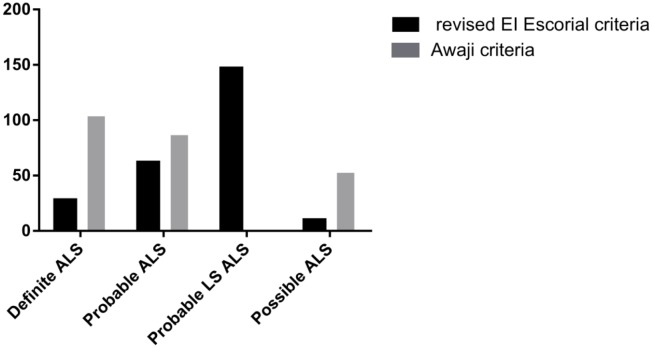
Diagnostic categories of the ALS patients according to the revised EI Escorial and Awaji criteria. Based on the rEEC, 4% were diagnosed as possible ALS, 60% as probable ALS-laboratory supported, 25% as probable ALS, and 11% as definite ALS. Following the application of the AC, the proportions were 22% possible ALS, 36% probable ALS and 42% definite ALS. ALS: amyotrophic lateral sclerosis; Probable LS: probable laboratory-supported.

The sensitivities of the probable or definite ALS diagnoses were 78% by the AC and 36% by the rEEC, and the difference was significant (*P* <0.001). Based on the rEEC, 4% were diagnosed as possible ALS, 60% as probable ALS-laboratory supported, 25% as probable ALS, and 11% as definite ALS. Following the application of the AC, the proportions were 22% possible ALS, 36% probable ALS and 42% definite ALS. The diagnostic categories of the ALS patients according to the AC and rEEC are illustrated in Fig 2. In the bulbar onset patients, diagnostic sensitivity increased from 39.1% to 76.1% by applying the AC, and it increased from 35.8% to 78.6% in the limb onset patients.

Table 1 displays the numbers of ALS patients in the different diagnostic categories. Using the AC, 70% (103/147) patients shifted from probable ALS-laboratory supported by the REEC to definite or probable ALS, and 30% (44/147) of the patients were demoted to possible ALS.

**Table 1:** Numbers of ALS patients in the different diagnostic criteria.

Revised EI Escorial criteria	N	Awaji criteria		
		Definite ALS	Probable ALS	Possible ALS
Possible ALS	10	0	0	10
Probable LS ALS	147	45	58	44
Probable ALS	62	32	30	0
Definite ALS	28	28	0	0
Total Patients	247	105	88	54

ALS: amyotrophic lateral sclerosis; Probable LS: probable laboratory-supported

## Discussion

In this study, we compared the diagnostic sensitivities of the AC and rEEC for ALS diagnosis in a Chinese population. We confirmed that the diagnostic sensitivity of the AC was significantly greater than that of the rEEC. Because ALS is a progressive and degenerative disease, early diagnosis and multidisciplinary management can help to improve the prognosis. Our results indicated that the AC may be more suitable for the diagnosis of ALS in clinical practice.

In total, 247 ALS patients were enrolled in our research; thus, this study is larger than any published report we could find [24, 25]. Carvalho et al (2009) collected 55 cases, and Gawel et al (2014) enrolled 135 ALS patients [9, 14]. Moreover, the study included patients in the early stage of ALS, and approximately 64% of the patients were diagnosed as clinically probable laboratory-supported ALS or clinically possible ALS using the rEEC.

The diagnostic sensitivities of clinically probable or definite ALS based on the rEEC in our study were lower than those reported in previous studies (55–59%) [12, 14, 22]. A possible reason for this discrepancy is that the patients in our study were in the early stage of ALS, and approximately 36% of the patients were clinically probably or definite ALS. In contrast, Chen et al (2010) identified 26 patients (57%) with clinically probably or definite ALS [12].

Compared with the rEEC, the AC includes additional changes. First, the EMG evidence of lower motor neuron degeneration is equal to clinical signs. Second, fasciculations are taken as equivalent to fibrillation potentials and positive sharp waves in terms of recognizing denervation [26], which is expected to increase the sensitivity for the diagnosis of ALS. Our study found that the AC indeed improved the diagnostic sensitivity for ALS (78% in the AC and the 36% in rEEC) and that the diagnostic sensitivity of the AC was similar to the sensitivities reported in previous studies (63–95%) [9, 14, 17, 27]. The results of our study in a Chinese population have strengthened the clinical evidence. Recently, in an attempt to increase sensitivity of the diagnostic criteria, Ludolphs et al [28] proposed a revision of the El Escorial criteria in which possible ALS was regarded as a positive finding. It can be concluded that progressive lower and upper motor neuron deficits in at least one region of the human body is sufficient for the diagnosis of ALS. The revision would ultimately increase the diagnostic sensitivity and provide the opportunity that possible ALS can be enrolled in clinical trials.

Clinically probable ALS-laboratory supported is in the early stage of ALS and involves fewer lesions [26], which has a significant effect on the diagnostic sensitivity. Based on the AC, 70% of the patients were shifted from clinically probable ALS-laboratory supported based on the rEEC to clinically definite or clinically probable ALS, and 30% of the patients were demoted to clinically possible ALS. The reason for the downgrades is that when only one upper motor neuron lesion and an electrophysiological record of two neurogenic lesions is present, the status is defined as clinically possible ALS based on the AC [7].

A limitation of the study is that the specificities of the two criteria were not investigated. The two criteria were designed to be highly specific, evidence of other diseases was excluded. In our study group, none of the 247 patients was false-positively diagnosed with ALS during follow-up. A possible explanation is the availability and application of additional diagnostic tests to exclude other ‘mimic disorders’ during diagnostic evaluation. Moreover, according to the recent meta-analysis confirming the increased sensitivity of Awaji criteria by Geevasinga N et al, [27]only four studies included a non-disease group and three out of the four studies reported a specificity of 100%; the fourth study reported a specificity of 80% but this was tested only a sample of 5 individuals(4/5). Although this was a single center-based study, the included data enrolled could to some extent reflect the characteristics of Chinese ALS patients.

In conclusion, the diagnostic sensitivity of the AC was significantly greater than that of the rEEC for an ALS diagnosis in a Chinese population. The application of the AC is recommended as the standard for the diagnosis of ALS in clinical practice.

## Supporting information

S1 File Original dataset. The original relevant data are within the paper.
(XLS)

S2 File STROBE_checklist_v4_combined_PlosMedicine. File contains the STROBE Checklist of this paper.
(DOCX)
